# The Clinical Value of Poor-Law Infirmaries

**Published:** 1915-03-06

**Authors:** 


### THE CLINICAL VALUE OF POOR-LAW INFIRMARIES.

The article we published under this heading in our Number of January 30 has aroused a considerable amount of interest amongst Poor-Law medical officers. A critical survey of the evidence we have received leads us to the conclusion that the clinical records of most of the large infirmaries under separate administration are, considering the admitted staff difficulty and the nature of Poor-Law work, not without system or value. As regards individual infirmaries there would appear to be, however, great differences in the value and fulness of the notes. In most they form an accurate and practical record of the patients' illness and treatment. In a minority they are meagre and barely fulfil the requirements of the Local Government Board's Orders. Much depends upon the energy of the medical superintendent, the amount of supervision he gives, and the value he attaches to clinical records. An important factor in the matter is the difficulty the Poor-Law infirmaries have in obtaining good medical officers—a difficulty due to the smallness of the salaries offered, to the understaffing of the infirmary, and to some odium which still attaches to the Poor-Law system. Another factor is the imperfect supervision of the Local Government Board, due to its lack of medical inspectors for Poor-Law purposes. In the analogous case of the Lunacy Board of Control, its medical members, when visiting an asylum, always inspect the note-books and report upon the way in which they are kept. Action of this kind has a most valuable stimulating effect and tends to the production of a uniformity of system in the keeping of the clinical records of the various asylums. A third factor is the smallness of the medical staff in the infirmaries. This of necessity leads to the notes being limited in most cases to the important points from a practical point of view, and to their being expressed in the most condensed form. This brevity of expression is not to be condemned, but is apt to mislead the inexperienced when comparing the infirmary records with those of our teaching hospitals. All of us who have had to read through the productions of clinical clubs at our hospitals know that the most voluminous notes are not always the most informative. Condensation has, however, this drawback—it leads to the main stress being laid upon positive findings and to the omission of negative results. Such omission often impairs the scientific value of the records, because if one is looking through a series of them for a specific purpose, it may be impossible to be sure that the absence of the record of a given symptom was due to the fact that it was not observed, or to the fact that it was not looked for.

This brings us to another aspect of the matter. The discussion in our columns has turned almost entirely upon the character of the clinical notes in the infirmaries. The larger matter, the utilisation of the clinical material contained in them, has hardly been touched upon. This material is even now being utilised in many ways. Our infirmaries afford an almost unique field in which many a newly qualified medical man has gained experience in all branches of his profession under conditions which have trained him in self-reliance and rapidity of decision. Cases of interest are often shown at meetings of medical societies. Medical practitioners avail themselves of the opportunity to consult with the medical staff on cases they send in and to follow their progress and treatment in the infirmary. The medical papers frequently contain important contributions from infirmary medical officers, and some of them have been entrusted with special investigations by the Local Government Board. Requests for the supply of pathological material and for facilities to carry out investigations are repeatedly received from the pathologist of the Local Government Board and from outside medical men and are willingly granted whenever possible. Nevertheless, the fact remains that research work in our infirmaries is not systematised and is not carried out to the extent it should be. One of the first fruits of a reorganisation of the Poor-Law Medical Service should be a great development in this respect. What is wanted is a special department, with adequate staff and laboratory facilities, to encourage research by special grants, by advice, by suggestion, and by the force of its example.

